# Polarization-Insensitive Electro-Optic Modulator for the Terahertz Regime Enabled by a Graphene-Hybrid Plasmonic Waveguide

**DOI:** 10.3390/nano16050288

**Published:** 2026-02-25

**Authors:** Xia Zhou, Caijing Liu, Yingting Li, Tingting Weng, Qilong Tan, Xuguang Huang, Jingshun Pan

**Affiliations:** 1 School of Optoelectronic Science and Engineering, South China Normal University, Guangzhou 510006, China; xzhou@m.scnu.edu.cn (X.Z.); 20223232085@m.scnu.edu.cn (C.L.); 2023022323@m.scnu.edu.cn (Y.L.); 2024022533@m.scnu.edu.cn (T.W.); 2 Institute of Semiconductors, Guangdong Academy of Sciences, Guangzhou 510075, China

**Keywords:** electro-optic modulator, graphene, polarization-insensitive, plasmonic waveguide

## Abstract

A polarization-insensitive compact optical modulator based on a graphene-hybrid surface plasmon polariton waveguide is proposed. The inverted U-shaped structure enables the synchronous control of TE/TM modes via Fermi level tuning, achieving a maximum attenuation of 0.247 dB/μm (E_f_ = 0.3 eV) and a minimum attenuation of 0.026–0.028 dB/μm (E_f_ = 1.0 eV) at 3 THz, with a polarization-dependent modulation error of only 0.002 dB/μm. The 100 μm × 30 μm device operates effectively at 2.5 THz (120 μm), demonstrating its potential for integrated photonic circuits. Additionally, the proposed modulator is compatible with Complementary Metal-Oxide-Semiconductor (CMOS) technology. The excellent ultra-broadband modulation performance of the graphene-hybrid plasmonic waveguide (GHPW) thereby paves the way for high-speed communication, non-destructive testing, biomedical sensing and optical computing.

## 1. Introduction

The rapid growth of data-intensive communication and sensing technologies has driven increasing demand for high-speed and broadband signal modulation. Electro-optic (EO) modulators play a key role in these systems by enabling efficient electrical-to-electromagnetic signal conversion and directly affecting metrics such as bit-error rate and signal-to-noise ratio [1,2,3,4,5]. While EO modulation has been extensively developed in the near-infrared (NIR) regime, integrated EO modulators operating in the terahertz (THz) band remain far less explored. The THz frequency range offers unique advantages for future sixth-generation (6G) communications, high-capacity wireless links, spectroscopy, and non-destructive imaging, owing to its wide available bandwidth and favorable penetration characteristics. However, the realization of efficient THz EO modulators faces several fundamental challenges that differ from those in optical systems, including the lack of suitable low-loss and high-efficiency EO materials, limited modulation efficiency, slow response speed, and strong polarization dependence caused by weak field confinement and waveguide dispersion [6,7]. Among these challenges, polarization sensitivity and waveguide mode control represent critical yet insufficiently addressed issues in THz modulation. Due to the wide operating bandwidth and low refractive-index contrast in the THz regime, guided modes are more susceptible to structural asymmetry and material anisotropy, which leads to enhanced polarization-dependent propagation characteristics. As a result, most reported THz modulators operate under a single polarization state, either transverse electric (TE) or transverse magnetic (TM), thereby limiting their applicability in polarization-multiplexed or multifunctional systems [8,9,10]. Graphene has emerged as a promising material platform for THz modulation owing to its gate-tunable surface conductivity and its ability to support surface plasmon polaritons (SPPs) in the mid-infrared to THz frequency range [11,12,13,14,15,16,17,18,19]. Graphene-hybrid plasmonic waveguides (GHPWs) enable strong electromagnetic confinement beyond the diffraction limit [20] and provide enhanced flexibility for mode engineering and polarization control. Nevertheless, existing graphene-based THz modulators are predominantly optimized for single-polarization operation, and polarization-insensitive modulation in the THz regime remains largely unexplored.

In this work, we propose a polarization-insensitive EO modulator operating in the terahertz frequency range based on a graphene-hybrid plasmonic waveguide. Unlike conventional EO modulators, the proposed device exploits the strong surface plasmon confinement enabled by graphene in the THz regime. By tuning the bias voltages applied to both sides of the graphene layer, the Fermi level of graphene can be continuously adjusted from 0.3 eV to 1.0 eV, enabling efficient excitation of different plasmonic modes with tunable propagation constants at the graphene/dielectric interfaces. It is worth noting that, under the same bias voltages, the propagation constants of excited SPPs around the graphene by different polarizations are unequal, and these small discrepancies compensate for the polarization-dependent modulation error caused by the structural asymmetry between horizontal and vertical directions. Thus, polarization-insensitive operation with low insertion loss is achieved within the target THz band.

The FDTD simulation results show that the MD difference in the GHPW between the TE and TM polarization modes is only 0.002 dB/μm. The MDs are 0.221 dB/μm for TE and 0.219 dB/μm for TM around the target frequency of 3 THz. This minimal discrepancy indicates the excellent polarization-insensitive performance and ultra-broadband characteristics of the modulator. These features pave the way for applications in high-speed communication, non-destructive testing, biomedical sensing, and optical computing.

## 2. Structure and Parameters

**Figure 1:**
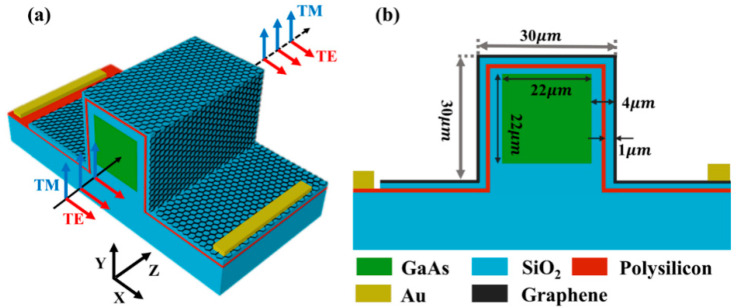
Structure diagram of the device. (**a**) A three-dimensional view of the waveguide; (**b**) a cross-sectional view and the specific parameters of the structure.

Figure 1 illustrates the structure of the graphene-based polarization-insensitive optical modulator. The modulator consists of a high-refractive-index GaAs core layer (*n* = 3.6) surrounded by a low-refractive-index silica cladding (*n* = 2.5), with two metal electrodes placed on top of graphene and polysilicon, respectively. The GaAs core layer has equal height and width of 22 μm. The silica cladding has a height and width of 30 μm. The spacing between the GaAs core layer and the top graphene, as well as between the two sidewall graphenes, is set at 4 μm. The number of graphene layers is one, with a thickness of 0.7 nm. The thickness of polysilicon (*n* = 1.732) is only 0.05 μm, which has a negligible effect on the mode profile distribution and transmission due to its proximity to the refractive index of silica. The polarization-insensitive performance of our graphene-assisted hybrid plasmonic waveguide originates from its symmetric dielectric configuration around the GaAs core. Since SPPs in graphene are exclusively excited by the surface-normal electric field component (E⊥), this geometric symmetry ensures balanced modulation of TE and TM modes. The emergence of antisymmetric surface plasmon polariton (SPP) modes in ultrathin graphene films is fundamentally attributed to its voltage-modulated quasi-metallic conduction behavior, where interfacial charge redistribution under externally applied electric fields induces strong intra-band transitions that dominate the electromagnetic mode hybridization process [21]. Under varying bias voltages, the two excited modes in this device exhibit identical evolution trends. At 0.3 eV, the excited modes correspond to antisymmetric modes localized on both sides of graphene, whereas at 0.7–1 eV, the modes become predominantly confined in the gap between GaAs and graphene. This distinct modal excitation mechanism under different biases enables the observed polarization-insensitive behavior.

The surface conductivity of graphene as a function of $\sigma_{\mathrm{total}}$ is explained below. According to the Kubo formula, the surface conductivity σ of graphene is composed of two parts: the inter-band ($\sigma_{\mathrm{inter}}$) and intra-band ($\sigma_{\mathrm{total}}$) terms [22].

(1) $$\sigma_{\mathrm{total}}=\sigma_{\mathrm{inter}}+\sigma_{\mathrm{intra}}$$

(2) $$\sigma_{\mathrm{intra}}=i\frac{e^{2}k_{B}T}{\pi \hbar^{2}(\omega +i\tau^{-1})}\left[\frac{\mu_{c}}{k_{B}T}+2\ln(e^{-\mu c/(k_{B}T)}+1)\right]$$

(3) $$\sigma_{\mathrm{inter}}=\frac{ie^{2}(\omega +i\tau^{-1})}{\pi \hbar^{2}}\int_{0}^{\infty }\frac{f_{d}(-\varepsilon )-f_{d}(\varepsilon )}{{\left(\omega +i\tau^{-1}\right)}^{2}-4{\left(\frac{\varepsilon }{\hbar }\right)}^{2}}d\varepsilon$$

Here, e is the electron charge, *ħ* is the reduced Planck constant, $k_{B}$ is the Boltzmann constant, ω is the angular frequency, T is the absolute temperature, τ is the momentum relaxation time, *μ_c_* is the chemical potential, and $f_{d}(\varepsilon )={\left[e^{\left(\varepsilon -\mu_{c})/(k_{B}T\right)}+1\right]}^{-1}$ is the Fermi-Dirac distribution. In the THz regime, where $k_{B}T\ll \mu_{c}$, the intra-band contribution dominates the conductivity, and the plasmon propagation constant and modal impedance are directly governed by the imaginary and real parts of $\sigma_{\mathrm{intra}}(\omega ,\mu_{c},\tau )$ respectively [23,24,25].

Therefore, it can be concluded that:

(4) $$\sigma_{\mathrm{intra}}=i\frac{e^{2}\mu_{c}}{\pi \hbar^{2}(\omega +i\tau^{-1})}$$

From the approximate formula above, it can be seen that the conductivity of graphene is related to frequency, scattering rate, temperature, and chemical potential. The chemical potential depends on the carrier density and can be adjusted by gate voltage, electric field, magnetic field, or chemical doping [26].

## 3. Simulation Results and Modulation Performance

We performed numerical simulations based on the finite-difference time-domain method, in which the boundary conditions of the designed graphene wave-guides were set as perfectly matched layers.

In this work, we systematically investigate the voltage-dependent attenuation [12] characteristics of both TE and TM polarization modes in graphene-based devices. We quantitatively define two key performance metrics: (i) insertion loss (corresponding to minimum attenuation) and (ii) MD (defined as the difference between maximum and minimum attenuation). Furthermore, the frequency-dependent attenuation behavior is comprehensively evaluated across the operational bandwidth.

### 3.1. Attenuation

**Figure 2:**
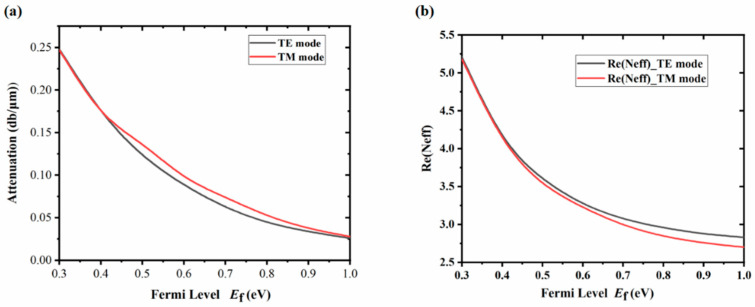
(**a**) Transmission attenuation and (**b**) effective refractive index vs. graphene Fermi level E_f_ for TE and TM modes at 3 THz.

In order to evaluate the modulation performance of the proposed GHPW optical modulator, we study the attenuation and Re(n_eff_) versus the graphene Fermi level. As shown in Figure 2a, the black curve illustrates the relationship between the transmission attenuation of the TE polarization mode and the Fermi level of graphene. The red curve shows the relationship between the transmission attenuation of the TM polarization mode and the Fermi level of graphene. Here, we set the frequency of incident light as f = 3 THz (λ~100 μm). As shown by the black curve, the transmission attenuation of the TE polarization mode decreases with the increase in the graphene Fermi level. When the graphene Fermi level is 0.3 eV, the TE polarization mode achieves a maximum transmission attenuation of 0.247 dB/μm. When the Fermi level of graphene is 1.0 eV, the TE polarization mode obtains the minimum transmission attenuation value of 0.026 dB/μm. Similarly, from the red curve, the transmission attenuation of the TM polarization mode decreases with the increase in the graphene Fermi level. When E_f_ is 0.3 eV, the maximum transmission attenuation value of the TM polarization mode is 0.247 dB/μm. When E_f_ is 1.0 eV, the minimum transmission attenuation value of the TM polarization mode is 0.028 dB/μm. In Figure 2a, the transmission attenuation of both TM and TE polarization modes shows the same variation with the graphene Fermi level. Under the same Fermi level, the transmission attenuation values of these two modes are similar. This close agreement between the two modes underscores the excellent polarization-insensitive modulation performance of the proposed modulator. When the graphene Fermi level is 0.3 eV, the transmission attenuation of TE and TM polarization modes is the largest at 0.247 dB/μm. We define E_f_ = 0.3 eV as the “OFF” state of the modulator. When the graphene Fermi level is 1.0 eV, the transmission attenuation of TE and TM polarization modes is the smallest, with values of 0.026 dB/μm and 0.028 dB/μm, respectively. We define E_f_ = 1.0 eV as the modulator’s “ON” state. At this time, the MD of the TE polarization mode is 0.221 dB/μm, and that of TM polarization mode is 0.219 dB/μm. The difference between the MDs of the two polarization modes is only 0.002 dB/μm. Therefore, we take 0.221 dB/μm as the MD of the polarization-insensitive modulator, and the insertion loss of the modulator is only 0.026 dB/μm. When the length of the graphene hybrid waveguide is 13.57 μm, a MD of 3 dB can be obtained, with an insertion loss of only 0.35 dB.

### 3.2. Effective Refractive Index

In Figure 2b, the black curve depicts the correlation between the real part of the effective refractive index of the TE polarization mode and the Fermi level of graphene. The red curve shows the relationship between the real part of the effective refractive index of the TM polarization mode and the Fermi level of graphene. As can be seen from the black curve, with the increase in graphene Fermi level, the real part of effective refractive index of the TE polarization mode gradually decreases. At the Fermi level E_f_ = 0.3 eV, the real part of the effective refractive index of the TE polarization mode reaches a maximum value of 5.21. When the Fermi level of graphene E_f_ = 1 eV, the real part of the effective refractive index of the TE polarization mode reaches a minimum value of 2.83. From the red curve, it can be seen that as the Fermi level of graphene increases, the real part of the effective refractive index of the TM polarization mode gradually decreases. When the Fermi level of graphene E_f_ = 0.3 eV, the maximum real part of the effective refractive index of the TM polarization mode is 5.18. When the Fermi level of graphene Ef = 1 eV, the real part of effective refractive index value of the TM polarization mode is at its minimum 2.70. From Figure 2b, we can see that the real parts of the effective refractive index of the TE and the TM polarization modes show a similar variation trend with the change in graphene Fermi level. When the Fermi level of graphene is the same, the real part of effective refractive index values of the two modes are almost identical.

### 3.3. Distributions of the Electric Field

**Figure 3:**
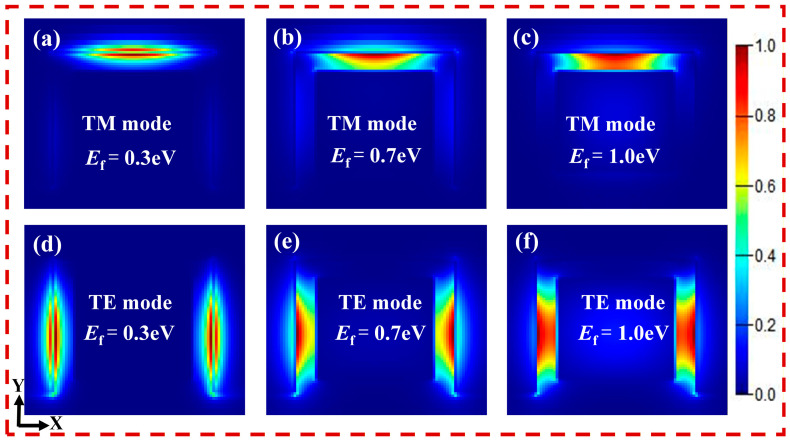
Field distributions on the transverse x-y cross-section at different E_f_. (**a**–**c**) TM polarization mode at E_f_ = 0.3 eV, 0.7 eV, and 1.0 eV, respectively; (**d**–**f**) TE polarization mode at E_f_ = 0.3 eV, 0.7 eV, and 1.0 eV, respectively.

Figure 3a, Figure 3b and Figure 3c are the electric field distributions of the TM polarization mode at E_f_ = 0.3 eV, 0.7 eV and 1.0 eV, respectively. Figure 3d, Figure 3e and Figure 3f are the electric field distributions of the TE polarization mode at E_f_ = 0.3 eV, 0.7 eV, and 1.0 eV, respectively. The THz wave propagates along the GaAs core, while its evanescent field penetrates into the thin SiO_2_ cladding and gradually decays. Owing to the subwavelength thickness of the SiO_2_ spacer, the evanescent field efficiently couples to the graphene layer and excites SPPs. These SPPs exhibit strong subwavelength confinement and significant field enhancement at the graphene interface. Consequently, the normalized field distribution in Figure 3 reveals higher field intensity near graphene than within the GaAs core. This pronounced energy concentration arises from plasmonic localization at the graphene interface, rather than from conventional dielectric confinement within the SiO_2_ cladding. From Figure 3a–c, we can see that as the Fermi level of graphene decreases, the electric field energy of the TM polarization mode is gradually becomes more concentrated towards the top graphene layer. When the graphene Fermi energy E_f_ = 1 eV, the electric field energy of the TM polarization mode is mainly concentrated in the silicon dioxide layer between the top graphene and core layer GaAs. At this time, the optical field energy bound near the top graphene is relatively small. The light absorption loss and transmission attenuation of the top graphene are low. However, the mode field area of the mode is relatively large, leading to a lower effective refractive index of the mode. As the Fermi level of graphene decreases to E_f_ = 0.7 eV, most of the optical field energy is bound near the top graphene. The effective area of the mode field is significantly reduced. This reduction leads to an increase in the absorption loss of the top graphene for light. Additionally, the transmission attenuation increases significantly. As the effective area of the mode field decreases, the effective refractive index of the mode increases. When the Fermi level of graphene is E_f_ = 0.3 eV, almost all the mode field energy is bound near the top graphene. This binding leads to a very large light absorption loss in the top graphene. Consequently, the mode loss is also very large. Additionally, the effective area of the mode field is very small, resulting in a relatively large effective refractive index of the mode. As shown in Figure 3d–f, as the Fermi level E_f_ of graphene decreases, the electric field energy of the TE polarization mode gradually becomes more concentrated in the graphene on both sides of the sidewall. The U-shaped configuration is extensively employed because it facilitates graphene cutting and enables efficient electrode fabrication. Moreover, its laterally symmetric geometry allows x-polarized light to excite SPPs on both sides of the graphene, as SPP excitation requires a perpendicular electric field component. As a result, the TE mode exhibits two symmetric field maxima in the cladding region. This symmetric field distribution is an inherent consequence of the U-shaped structure and is absent in L-shaped configurations, where only one-sided SPP excitation can occur. When the Fermi level of graphene E_f_ = 1 eV, the electric field energy of the TE polarization mode is mainly concentrated in the low-refractive-index silicon dioxide layer between the sidewall graphene and the high-refractive-index GaAs core layer. The energy bound in the vicinity of the sidewall graphene on both sides is relatively small. Thus, the absorption loss of the sidewall graphene is relatively low.

The transmission attenuation is relatively small. However, the mode field area of the mode is relatively large, leading to a relatively low effective refractive index of the mode. As the Fermi level of graphene decreases to E_f_ = 0.7 eV, most of the optical field energy is bound near the sidewall of graphene. The effective area of the mode field is significantly reduced. This reduction results in an increase in the absorption loss of the sidewall graphene. Additionally, the transmission attenuation increases significantly. As the effective area of the mode field decreases, the effective refractive index of the mode increases. When the Fermi level of graphene is E_f_ = 0.3 eV, almost all the mode field energy is bound near the sidewall of graphene. This binding results in very large light absorption loss and mode loss by the sidewall graphene. The effective area of the mode field is small, leading to a relatively large effective refractive index of the mode. The strong modal extension into the cladding region increases the effective impedance of the hybrid plasmonic section compared to the dielectric input waveguide. However, this effect does not degrade the modulation performance. As the mode displacements are much stronger at the “OFF” state, the introduced loss is beneficial for improving the modulation depth. And, due to the small size of effective modulation area (≤100 μm), the loss is controllable at the “On” state. Therefore, the impedance variation associated with modal redistribution does not significantly compromise device performance.

### 3.4. Operating Bandwidth

**Figure 4:**
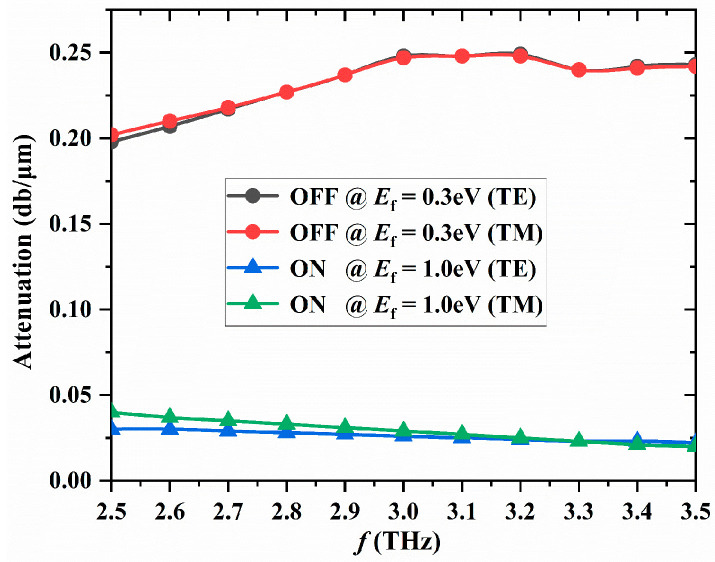
Transmission attenuation of TE and TM modes under the modulator “OFF” and “ON” states at different frequencies.

In addition to the MD and the insertion loss, the operating bandwidth is also an important factor in evaluating the performance of a modulator. Our proposed graphene polarization-insensitive modulator operates effectively in the 2.5 THz to 3.5 THz frequency range, achieving a bandwidth of 1 THz. As shown in Figure 4, when the Fermi level of graphene is E_f_ = 0.3 eV (OFF), the black curve represents the transmission attenuation in TE polarization mode across the 2.5 THz to 3.5 THz frequency range. The red curve represents the transmission attenuation of the TM polarization mode across the 2.5 THz to 3.5 THz frequency range. From the two curves, the transmission attenuations of the TE and TM polarization modes are very close and share the same change trend. In the frequency range of 2.5 THz to 3.5 THz, when the Fermi level of graphene is E_f_ = 0.3 eV (OFF state), the transmission attenuation of both the TE and TM polarization modes remains nearly constant at approximately 0.22 dB/μm. When f = 3 THz, the maximum transmission attenuation of the two polarization modes is 0.248 dB/μm. When the Fermi level of graphene is E_f_ = 1.0 eV (ON state), the blue curve represents the transmission attenuation of the TE polarization mode in the 2.5 THz to 3.5 THz frequency range. The green curve represents the transmission attenuation of the TM polarization mode in the 2.5–3.5 THz frequency range. From these two curves, it can be observed that the transmission attenuations of the TE and TM polarization modes are also very close and have the same trend. In the 2.5 THz to 3.5 THz frequency range, the transmission attenuation of TE and TM polarization modes does not change significantly at the Fermi level of graphene E_f_ = 0.3 eV (OFF state), remaining around 0.025 dB/μm. When f = 3.5 THz, the transmission attenuation of both polarization modes is at its minimum 0.02 dB/μm. It can be clearly seen from Figure 4 that in the entire frequency range of 2.5 THz to 3.5 THz, both the TE and TM polarization modes have very close transmission attenuation values, regardless of whether they are in the OFF or ON state. This further demonstrates that the proposed graphene polarization-insensitive modulator we designed exhibits excellent modulation capability in the entire frequency range of 2.5 THz to 3.5 THz. By calculation, in the frequency range of 2.5 THz to 3.5 THz, the MD of the polarization-insensitive modulator is greater than 0.2 dB/μm, and the insertion loss (IL) is below 0.02 dB/μm.

### 3.5. Transmission Process of the Electric Field Under the Modulator “OFF” and “ON”

**Figure 5:**
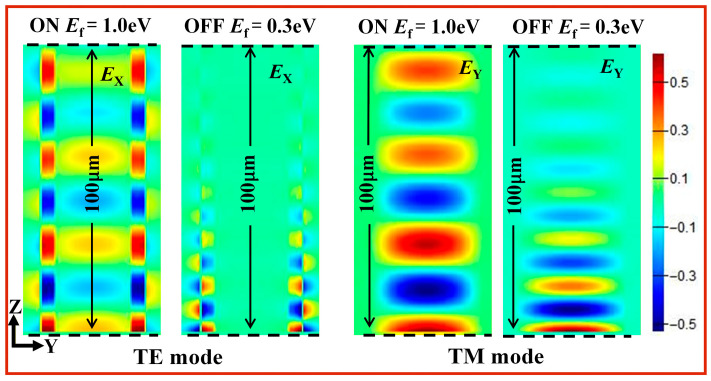
Field distributions on the longitudinal y–z cross-section. Transmission of the electric field E_X_(TE)/E_Y_(TM) along the *z*-direction for TE polarization mode and TM polarization mode in the E_f_ = 1.0 eV (ON) state and the E_f_ = 0.3 eV (OFF) state under the incident light frequency f = 3 THz.

Figure 5 shows the transmission process of the electric field E_X_(TE)/E_Y_(TM) along the z direction for the TE and TM polarization modes in the “ON” state (E_f_ = 1.0 eV) and the “OFF” state (E_f_ = 0.3 eV) when the frequency of the incident light is 3 THz. We can clearly see that when the Fermi level of graphene is E_f_ = 1.0 eV (ON), the electric field EX of the TE polarization mode experiences very little loss during the entire 100 μm transmission process. The transmission attenuation of the TE polarization mode is only 0.026 dB/μm at the Fermi level of graphene E_f_ = 1.0 eV. For a transmission length of 100 μm, the loss is only 2.6 dB. However, when the Fermi level of graphene is E_f_ = 0.3 eV (OFF), the transmission attenuation of TE polarization mode is very large, at 0.248 dB/μm. As shown in Figure 5, the electric field energy of the TE polarization mode is almost zero when the transmission length is 50 μm, resulting in a transmission attenuation of 12.4 dB over 50 μm. Similarly, when the Fermi level of graphene is E_f_ = 1.0 eV (ON), the electric field E_Y_ of the TM polarization mode remains very small throughout the entire 100 μm transmission process. Because the transmission attenuation of the TM polarization mode at the Fermi level E_f_ = 1.0 eV is only 0.028 dB/μm, the loss for a 100 μm transmission length is only 2.8 dB. However, when the Fermi level of graphene E_f_ = 0.3 eV is “OFF”, the transmission attenuation of the TM polarization mode is very large, at 0.247 dB/μm. As can be seen from the figure, when the transmission length is 50 μm, the electric field energy of the TM polarization mode is almost zero. The transmission attenuation is 12.35 dB over 50 μm. The transmission processes of the two modes shown in Figure 5 at the “OFF” and “ON” states are a good demonstration of the excellent modulation capability of the graphene waveguide we designed.

Finally, to better illustrate the characteristics of the proposed device, the simulation results of the reported polarization-insensitive optical modulator are compared with those obtained from this GHPW optical modulator. As listed in Table 1 below, the optical modulator proposed herein has three advantages: (1) The GHPW enables a compact structure. (2) The presented modulator has a relatively wide bandwidth, operating in the terahertz band (2.5 THz to 3.5 THz). (3) The proposed modulator possesses lower MD and IL.

## 4. Conclusions

In this work, a polarization-insensitive optical modulator based on a graphene-hybrid plasmonic waveguide is proposed for terahertz applications. The device utilizes an inverted “U”-shaped graphene structure and applied voltage to synchronously control the transmission attenuation of both TE and TM polarization modes. Numerical simulations demonstrated that the transmission attenuation for both modes increases as graphene Fermi level decreases. At E_f_ = 0.3 eV and f = 3 THz, the maximum transmission attenuation for TE and TM modes is 0.247 dB/μm. At E_f_ = 1.0 eV, the minimum transmission attenuations are 0.026 dB/μm and 0.028 dB/μm for TE and TM modes, respectively, resulting in an exceptionally low polarization-dependent modulation error of only 0.002 dB/μm. Furthermore, at an operating wavelength of 120 μm (corresponding to 2.5 THz), the modulator boasts an ultra-compact footprint of 100 μm × 30 μm, emerging as a promising core candidate for future integrated terahertz systems—with applications spanning high-speed communications, underwater communication networking, non-destructive testing, biomedical sensing, and optical computing.

## Figures and Tables

**Table 1:** Comparison of modulation performance.

Scheme	Δλ/λ	MD (dB)	IL (dB)	Footprint (μm^2^)
NPSOMS [27]	0.0219	-	1.61 dB	-
GMR [28]	0.0253	-	-	50 × 50
ITO-D1 [29]	0.1935	-	1.65 dB	34 × 1.875
PIM [30]	0.0645	>17 dB	<5.7 dB	-
DBOSW [31]	0.5161	>9.24 dB	-	20 × 0.48
EAM [22]	0.1942	>22 dB	<0.23 dB	20 × 0.6
PIGM [10]	0.5161	>10.04 dB	-	~20 × 0.34
PEM [32]	0.0194	-	4 dB	-
EAPM [23]	0.0226	3 dB	2 dB	50 × 0.407
This work	0.7100	>2.19 dB	<2.8 dB	100 × 30

## Data Availability

The original contributions presented in this study are included in the article. Further inquiries can be directed to the corresponding authors.

## References

[1] Yan Y., Zhang H., Liu X., Peng L., Zhang Q., Yu G., Wu Q., Li H. (2025). Recent Progress in Electro-Optic Modulators: Physical Phenomenon, Structures Properties, and Integration Strategy. Laser Photonics Rev..

[2] Zhang Y., Xiao X., Chen J., Zhang H., Huang Y., Si J., Liang S., Xu Q., Zhang H., Ma L. (2025). Lithium Niobate Crystal Preparation, Properties, and Its Application in Electro-Optical Devices. Inorganics.

[3] Wang J., Luo H., Meng Z., Hu Y. (2012). Experimental Research of an All-Polarization-Maintaining Optical Fiber Vector Hydrophone. J. Light. Technol..

[4] Guan B.O., Jin L., Zhang Y., Tam H.Y. (2012). Polarimetric heterodyning fiber grating laser sensors. J. Light. Technol..

[5] Guan B.O., Tan Y.N., Tam H.Y. (2009). Dual polarization fiber grating laser hydrophone. Opt. Express.

[6] Tonouchi M. (2007). Cutting-edge terahertz technology. Nat. Photonics.

[7] Nagatsuma T., Ducournau G., Renaud C.C. (2016). Advances in terahertz communications accelerated by photonics. Nat. Photonics.

[8] Huang B.H., Lu W.B., Li X.B., Wang J., Liu Z.G. (2016). Waveguide-coupled hybrid plasmonic modulator based on graphene. Appl. Opt..

[9] Ye S.-W., Liang D., Lu R.-G., Shah M.K., Zou X.-H., Yuan F., Yang F., Liu Y. (2016). Polarization-independent modulator by partly tilted graphene-induced electro-absorption effect. IEEE Photonics Technol. Lett..

[10] Liu S., Wang M., Liu T., Xu Y., Yue J., Yi Y., Sun X., Zhang D. (2022). Polarization-Insensitive Graphene Modulator Based on Hybrid Plasmonic Waveguide. Photonics.

[11] Novoselov K.S., Geim A.K., Morozov S.V., Jiang D., Zhang Y., Dubonos S.V., Grigorieva I.V., Firsov A.A. (2004). Electric field effect in atomically thin carbon films. Science.

[12] Geim A.K., Novoselov K.S. (2009). The rise of graphene. Nat. Mater..

[13] Bonaccorso F., Sun Z., Hasan T., Ferrari A.C. (2010). Graphene photonics and optoelectronics. Nat. Photonics.

[14] Liao L., Lin Y.C., Bao M., Cheng R., Bai J., Liu Y., Qu Y., Wang K.L., Huang Y., Duan X. (2010). High-speed graphene transistors with a self-aligned nanowire gate. Nature.

[15] Avouris P., Chen Z., Perebeinos V. (2007). Carbon-based electronics. Nat. Nanotechnol..

[16] Tan Q., Guo Q., Liu H., Huang X., Zhang S. (2017). Controlling plasmonic orbital angular momentum by combining geometric and dynamic phases. Nanoscale.

[17] Tan Q., Xu Z., Zhang D.H., Yu T., Luo Y. (2020). Polarization-Controlled Plasmonic Structured Illumination. Nano Lett..

[18] Wang H., Yang N., Chang L., Zhou C., Li S., Deng M., Li Z., Liu Q., Zhang C., Li Z. (2020). CMOS-compatible all-optical modulator based on the saturable absorption of graphene. Photonics Res..

[19] Pospischil A., Humer M., Furchi M.M., Bachmann D., Guider R., Fromherz T., Mueller T. (2013). CMOS-compatible graphene photodetector covering all optical communication bands. Nat. Photonics.

[20] Low T., Avouris P. (2014). Graphene Plasmonics for Terahertz to Mid-Infrared Applications. ACS Nano.

[21] Dionne J.A., Sweatlock L.A., Atwater H.A., Polman A. (2005). Planar metal plasmon waveguides: Frequency-dependent dispersion, propagation, localization, and loss beyond the free electron model. Phys. Rev. B.

[22] Xu Y., Li F., Kang Z., Huang D., Zhang X., Tam H.Y., Wai P. (2019). Hybrid Graphene-Silicon Based Polarization-Insensitive Electro-Absorption Modulator with High-Modulation Efficiency and Ultra-Broad Bandwidth. Nanomaterials.

[23] Hu X., Wang J. (2018). Design of graphene-based polarization-insensitive optical modulator. Nanophotonics.

[24] Gan C.H., Chu H.S., Li E.P. (2012). Synthesis of highly confined surface plasmon modes with doped graphene sheets in the midinfrared and terahertz frequencies. Phys. Rev. B.

[25] Cui L., Wang J., Sun M. (2021). Graphene Plasmon for Optoelectronics. Rev. Phys..

[26] Yi J. (2021). Polarization-Insensitive Broadband THz Absorber Based on Circular Graphene Patches. Nanomaterials.

[27] Liang W., Chen W., Wang P., Dai S., Yang J. (2020). Non-volatile polarization-insensitive 1 × 2 silicon optical switch using phase-change materials. Opt. Commun..

[28] Ju Y., Zhang W., Zhao Y., Deng X., Zuo H. (2024). Polarization independent lithium niobate electro-optic modulator based on guided mode resonance. Opt. Mater..

[29] Abdelatty M.Y., Badr M.M., Swillam M.A. (2018). Compact Silicon Electro-Optical Modulator Using Hybrid ITO Tri-Coupled Waveguides. J. Light. Technol..

[30] Yang Z., Lu R., Cai S., Wang Y., Liu Y. (2019). A CMOS-compatible and polarization-insensitive graphene optical modulator. Opt. Commun..

[31] Chen W., Xu Y., Gao Y., Ji L., Zhang D. (2021). A Broadband Polarization-Insensitive Graphene Modulator Based on Dual Built-in Orthogonal Slots Plasmonic Waveguide. Appl. Sci..

[32] Liu X., Chen N., Chu T. (2024). Polarization-Insensitive Electrooptic Modulation on Anisotropic Thin-Film Lithium Niobate. ACS Photonics.

